# Association between ratio of measured extracellular volume to expected body fluid volume and renal outcomes in patients with chronic kidney disease: a retrospective single-center cohort study

**DOI:** 10.1186/1471-2369-15-189

**Published:** 2014-12-01

**Authors:** Reibin Tai, Yasushi Ohashi, Sonoo Mizuiri, Atsushi Aikawa, Ken Sakai

**Affiliations:** Department of Nephrology, School of Medicine, Faculty of Medicine, Toho University, 6-11-1 Omori-Nishi, Ota-ku, Tokyo, 143-8541 Japan; Division of Nephrology, Ichiyokai Harada Hospital, Hiroshima, Japan

**Keywords:** Chronic kidney disease, Extracellular volume excess, Kidney disease progression

## Abstract

**Background:**

Excess extracellular volume is a major clinical problem in patients with chronic kidney disease (CKD). However, whether the extracellular volume status is associated with disease progression is unclear. We investigated the association between the extracellular volume status and renal outcomes.

**Methods:**

We performed a retrospective cohort study of 149 patients with CKD who underwent bioelectrical impedance analysis (BIA) from 2005 to 2009. Patients were categorized according to tertiles of extracellular volume status. The extracellular volume status was assessed by examining the ratio of extracellular water measured by BIA (ECW_BIA_) to the total body water calculated using the Watson formula (TBW_Watson_). The main outcomes were adverse renal outcomes as defined by a decline of ≥50% from the baseline glomerular filtration rate or initiation of renal replacement therapy.

**Results:**

A higher %ECW_BIA_/TBW_Watson_ ratio tended to be associated with older age, male sex, diabetes mellitus, resistant hypertension, lower renal function, lower serum albumin levels, higher proteinuria levels, and a higher frequency of furosemide use. In the multivariate analysis, proteinuria remained independently associated with the %ECW_BIA_/TBW_Watson_ ratio. Both the intracellular and extracellular water volumes decreased with age (correlation between ICW and age, *r* = -0.30, *P* < 0.001; correlation between ECW and age, *r* = -0.17, *P* = 0.03). Consequently, the %ECW_BIA_ in the body fluid composition increased with age. During a median follow-up of 4.9 years, patients in the highest tertile of the %ECW_BIA_/TBW_Watson_ ratio were at greater risk of adverse renal outcomes (16.6 per 100.0 patient years) than were those in the lowest tertile (8.1 per 100.0 patient years) or second tertile (5.6 per 100.0 patient years) (log-rank *P* = 0.005). After adjustment for covariates, the %ECW_BIA_/TBW_Watson_ ratio was significantly associated with adverse renal outcomes (hazard ratio, 1.21; 95 % confidence interval, 1.10–1.34; *P* < 0.001).

**Conclusions:**

The ECW_BIA_/TBW_Watson_ ratio was independently associated with adverse renal outcomes. Proteinuria was independently associated with the extracellular volume status. The balance between ICW and ECW changes with age in that the percentage of ECW content in the body fluid composition increases. Elderly patients with CKD may thus be susceptible to volume overload.

**Electronic supplementary material:**

The online version of this article (doi:10.1186/1471-2369-15-189) contains supplementary material, which is available to authorized users.

## Background

Excess extracellular volume is a major clinical problem in patients with chronic kidney disease (CKD) and causes lower extremity edema, hypertension, pulmonary vascular congestion or edema, and heart failure [1]. However, whether excess extracellular volume is associated with kidney disease progression is unclear, and such studies are hampered by the lack of suitable markers of hypervolemia. In bioimpedance analysis (BIA), the ratio of extracellular water (ECW) to total body water (TBW) has been used as an indicator of the fluid volume status [2–6]. However, the ECW/TBW ratio may not be an ideal measurement of volume overload. The ECW/TBW ratio is affected not only by changes in the ECW, but also by changes in the intracellular water (ICW) component of TBW. The amount of ICW decreases with aging [7]. Therefore, an increased ECW/TBW ratio may be also observed in lean, elderly patients without edema. Using the Body Composition Monitor, which assesses the extracellular volume status by comparing the measured ECW to the expected ECW, Tsai et al. recently reported an association between fluid overload and adverse kidney outcomes in the short term in patients with advanced stage 4 to 5 CKD [8].

Anthropometric formulas combined with tracer dilution techniques have been extensively used to calculate TBW in patients with CKD. The Watson formula, which is routinely used to calculate the efficiency of dialysis, was originally derived from pooled data of healthy subjects and provides information about adequate fluid volume adjusted for age and sex [9]. We hypothesized that the ratio of the ECW as measured by BIA (ECW_BIA_) to the TBW as calculated using the Watson formula (TBW_Watson_) might also be used to assess the extracellular fluid status in clinical practice. In addition, we assessed the extracellular volume status using the ratio of the measured ECW to the expected ECW as calculated by an equation described by Peters et al. based on healthy potential renal transplant donors [10].

The goals of the present study were to (1) identify factors associated with the extracellular volume status, (2) investigate the relationship between the extracellular volume status and renal outcomes in patients with CKD and n study the prognostic performance of parameters associated with the extracellular volume status in predicting adverse renal outcomes.

## Methods

### Study design

This study was approved by the Ethics Committee of Toho University Omori Medical Center, Tokyo, Japan (approval number: 25–252) and was performed in adherence with the Declaration of Helsinki. Informed consent was obtained from all participants. Of 170 patients with CKD aged ≥20 years with BIA body composition measurements obtained from August 2005 to January 2009, 149 patients with complete clinical data in whom we could assess anthropometric measurements, blood pressure, proteinuria, and kidney function at the same time were studied.

The following patient characteristics and parameters were examined: age, sex, height, body weight, body mass index (BMI), underlying renal disease, office blood pressure, serum albumin level, total cholesterol level, triglyceride level, fasting blood glucose level, uric acid level, serum creatinine (Cr) level, estimated glomerular filtration rate (eGFR), urinary protein to creatinine ratio in a random urine sample (UPCR), and use of diuretics and antihypertensive agents. In total, 127 of the 147 patients were able to provide a 24-hour urine sample. Hyperuricemia was diagnosed if the uric acid level was >7.0 mg/dL for men and >5.7 mg/dL for women in accordance with previously performed population surveys [11]. The eGFR was calculated according to the CKD-Epidemiology Collaboration equation [12]. Resistant hypertension was defined as uncontrolled blood pressure (office systolic blood pressure of ≥130 mmHg or office diastolic blood pressure of ≥80 mmHg) despite antihypertensive therapy using three or more medications, including diuretics. Controlled blood pressure using four or more drugs was also defined as resistant hypertension [13, 14]. Patients were followed up until August 2013 (median, 1789 days; 10th–90th percentile, 422–2689 days) unless loss to follow-up or death occurred.

### Adverse outcomes

Cox proportional hazards models with time-dependent covariates were used to compare renal outcomes and all-cause mortality. The endpoint of the study was the time to the first record of either one of the following adverse events: ≥50% decline in the GFR relative to baseline or initiation of either dialysis therapy or renal transplantation [15, 16].

### Assessment of body fluid status

BIA was performed in a standard manner with the patient lying supine on a flat, nonconductive bed for at least 15 min. A segmental BIA instrument (Inbody S20®; Biospace Co., Ltd., Seoul, Korea [http://www.biospaceamerica.com]) with eight tactile electrodes was used. The microprocessor-controlled switches and BIA analyzer were activated, and segmental resistances of the arms, trunk, and legs were measured at four frequencies (5, 50, 250, and 500 kHz). Thus, 20 segmental resistances were measured for each patient. Using the BIA software, the sum of the segmental resistances for each body segment was used to calculate the TBW, ICW, and ECW. Each measured fluid compartment was expressed as both the actual value and percentage of the body weight. We calculated the expected ECW according to the Peters formula (ECW_Peters_): (-2.47 × 0.842 + 8.76 × body surface area) for men and (-1.96 × 0.572 + 8.05 × body surface area) for women [10]. We also estimated the TBW according to the Watson formula (TBW_Watson_): (2.447 + [0.0956 × age] + [0.1074 × height] + [0.3362 × body weight]) for men and (-2.097 + [0.1069 × height] + [0.3362 × body weight]) for women [9]. The body surface area was estimated using the equation described by Haycock et al. [17]. The ratio of ECW_BIA_ to ECW_Peters_ or the percentage of ECW_BIA_ to TBW_Watson_ were modified as indicators of excess fluid volume [7].

### Statistical analyses

Data were statistically analyzed using JMP 9.0 software (SAS Institute, Inc., Cary, NC, USA). Patients were classified into tertiles according to their extracellular volume status. Because all parameters used to assess the extracellular volume status had different actual values and percentages between men and women, we divided the tertile values by men and women and combined tertiles 1, 2, and 3 in men and women, respectively. Each measured value was expressed as either mean ± standard deviation or percentage. Statistical significance was assessed using a linear regression model to compare the mean values of possible risk factors among the tertile groups [18] and were checked by one-way analysis of variance for continuous variables and Pearson’s chi-squared test for categorical variables. We constructed Bland–Altman and residual plots of TBW_BIA_ and TBW_Watson_ to assess bias. Correlations between variables were examined using Pearson’s product–moment correlation coefficient. Logistic and linear regression analyses were used to analyze the association between the %ECW_BIA_/TBW_Watson_ ratio and demographic factors. Explanatory variables that were significantly correlated (*P* < 0.1) with the %ECW_BIA_/TBW_Watson_ ratio were then subjected to multivariate analysis to identify independent associations. Kaplan–Meier survival curves for adverse renal outcomes were generated. A Cox regression model with time-dependent covariates was used to analyze the relationship between the %ECW_BIA_/TBW_Watson_ ratio and adverse renal outcomes, and analyzed values were expressed as hazard ratios (HRs) with corresponding 95% confidence intervals (CIs). Receiver operating characteristic curve analysis was used to identify the best prognostic factor for adverse renal outcomes. A probability (*P*) value of <0.05 was considered to be statistically significant.

## Results

### Comparison between percentage of ECW in body weight, ratio of ECW_BIA_ to ECW_Peters_, and percentage of ECW_BIA_ to TBW_Watson_

**Figure 1:**
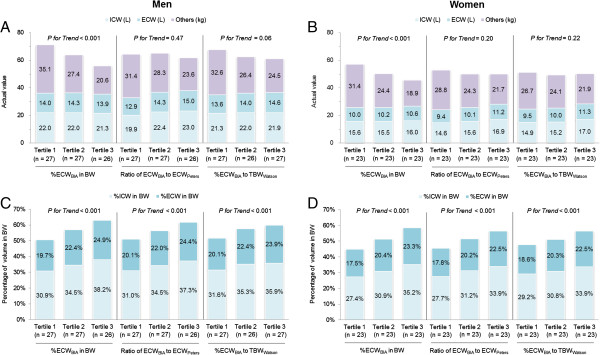
**Associations between extracellular volume status and actual values of body composition (A in men and B in women) and percentages of fluid volume in body weight (C in men and D in women).** Abbreviations: BW, body weight; ECW_BIA_, extracellular water as measured by electrical bioimpedance analysis; ECW_Peters_, extracellular water calculated using the Peters formula; TBW_Watson_, total body water calculated using the Watson formula.

We compared three parameters of extracellular volume status assessment: (%ECW_BIA_ in body weight, ECW_BIA_/ECW_Peters_ ratio, and %ECW_BIA_/TBW_Watson_ ratio). As shown in Figure 1, patients with a higher %ECW_BIA_ were more likely to have a lower body weight. The %ECW_BIA_ in body weight was negatively correlated with body weight regardless of the actual extracellular volume status. On the other hand, patients in a higher tertile with respect to the other two parameters were more likely to have both higher actual values and higher extracellular volume percentages. We also observed preliminarily correlations between these three parameters and the patients’ main demographic characteristics (Additional file 1: Table S1). The %ECW_BIA_ in body weight was higher among patients with lower body weights and was only correlated with the serum albumin level. The ECW_BIA_/ECW_Peters_ ratio was positively correlated with a higher prevalence of resistant hypertension and furosemide use, lower serum albumin level, and higher UPCR level; it was also highly dependent upon height, explaining why height was included in the equation described by Peters et al. [10]. The %ECW_BIA_/TBW_Watson_ ratio exhibited a significant correlation with most demographic factors among these three parameters. Interestingly, the ECW_BIA_/ECW_Peters_ ratio and the %ECW_BIA_/TBW_Watson_ ratio showed different associations with age. Age tended to increase as the ECW_BIA_/ECW_Peters_ ratio decreased, whereas age tended to increase as the %ECW_BIA_/TBW_Watson_ ratio increased. This difference may be explained by the fact that ECW_BIA_ decreased with age in our population but that ECW_Peters_ did not decrease with age in a potential healthy donor population [10]. For the above-mentioned reasons, we used the %ECW_BIA_/TBW_Watson_ ratio as the main parameter of the extracellular volume status.

### Correlations between age and an imbalance between measured ICW and ECW

**Figure 2:**
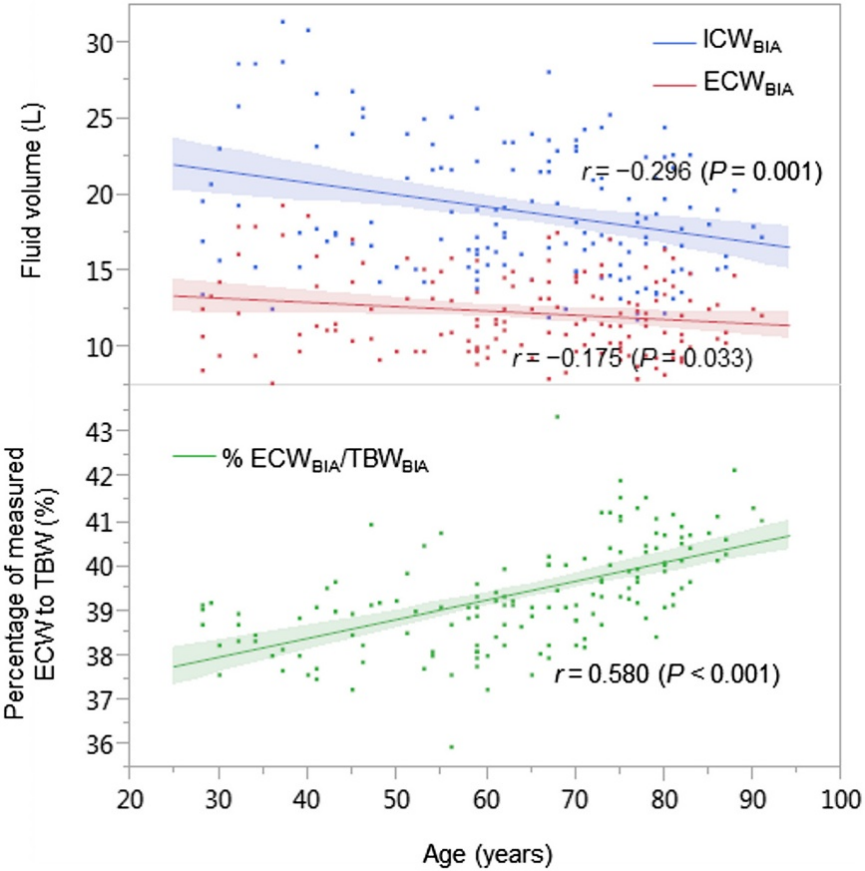
**Correlations between age with an imbalance between ICW and ECW.** Abbreviations: ICW_BIA_, intracellular water; ECW_BIA_, extracellular water; TBW_BIA_, total body water as measured by bioimpedance analysis.

Correlations between age and an imbalance between the measured ICW and ECW are presented in Figure 2. Both the ICW and ECW content decreased with age, although the trend was predominantly observed in the ICW content (correlation between ICW and age, *r* = -0.30, *P* = 0.001; correlation between ECW and age, *r* = -0.18, *P* = 0.03). Thus, the percentage of measured ECW_BIA_ in TBW_BIA_ had a moderately positive correlation with age (*r* = 0.60, *P* < 0.001).

### Relationship between measured TBW_BIA_ and estimated TBW_Watson_ by sex

The mean differences between the measured TBW_BIA_ and estimated TBW_Watson_ for men and women were 0.41 L (95% CI, -0.19 to 1.01; *P* = 0.17) and -0.75 L (95% CI, -1.27 to -0.23; *P* = 0.01), respectively (Additional file 2: Figure S1A and C). Residual plots showed that bias in the measured TBW_BIA_ and estimated TBW_Watson_ were greatest at higher measured TBW_BIA_ values for both sexes (Additional file 2: Figure S1B and D).

### Patient characteristics at the time of BIA

The characteristics of the 149 patients according to the tertiles of the %ECW_BIA_/TBW_Watson_ ratio are listed in Table 1. The tertile values were 38.3% and 41.1% in men and 37.0% and 39.4% in women. Of the 149 patients, 80 were men and 69 were women, with a mean age of 63.7 ± 16.1 years; 24 patients (16.1%) had diabetes mellitus. The mean eGFR was 63.8 ± 50.1 ml/min per 1.73 m^2^. Patients with a higher %ECW_BIA_/TBW_Watson_ ratio tended to have diabetes mellitus, resistant hypertension, a lower diastolic blood pressure, a higher pulse pressure, a higher serum creatinine level, a lower eGFR, a lower serum albumin level, a higher UPCR level, and a higher prevalence of furosemide use (*P* < 0.05). In contrast, there were no significant differences in the 24-hour urinary sodium excretion rate among the three groups. Patients in the lowest tertile were more likely to have a higher BMI than were those in the higher tertiles (24.0 ± 4.2 kg/m^2^ in the lowest tertile vs. 22.0 ± 3.5 kg/m^2^ in the second tertile and 21.6 ± 2.9 kg/m^2^ in the highest tertile, *P* = 0.001). Notably, patients in the higher tertiles of the %ECW_BIA_/TBW_Watson_ ratio were more likely to have a higher percent body weight of TBW, ICW, and ECW.

**Table 1:** **Patients characteristics separated by tertiles of the percentage of ECW**_**BIA**_ **to TBW**_**Watson**_

Population characteristics	Percentage of ECW _BIA_to TBW _Watson_			***P***for ANOVA
	Tertile 1 (***n*** = 50) (27 men with <38.3% and 23 women with <37.0%)	Tertile 2 (***n*** = 49) (26 men with 38.3%–41.0% and 23 women with 37.0%–39.4%)	Tertile 3 (***n*** = 50) (27 men with ≥41.1% and 23 women with ≥39.5%)	
Age, years	62.0 ± 16.6	61.8 ± 15.1	67.1 ± 16.4	0.18
Height, cm	159 ± 10	159 ± 11	161 ± 8	0.46
Weight, kg	61 ± 15	56 ± 12	56 ± 10	0.09
Body surface area, m^2^	1.62 ± 0.21	1.56 ± 0.21	1.58 ± 0.16	0.41
Body mass index, kg/m^2^	24.0 ± 4.2	22.0 ± 3.5	21.6 ± 2.9	<0.01
*Underlying disease*				
Glomerulonephritis, *n* (%)	23 (46.0)	22 (45.0)	17 (34.0)	0.41
Diabetes mellitus, *n* (%)	4 (8.0)	7 (14.3)	13 (26.0)	<0.05
Nephrosclerosis, *n* (%)	9 (18.0)	10 (20.4)	9 (18.0)	0.94
ADPKD, *n* (%)	0 (0.0)	0 (0.0)	1 (2.0)	0.37
Others, *n* (%)	14 (28.0)	10 (20.4)	10 (20.0)	0.56
Systolic blood pressure, mmHg	127 ± 19	122 ± 17	129 ± 21	0.19
Diastolic blood pressure, mmHg	75 ± 11	71 ± 9	69 ± 10	<0.01
Pulse pressure, mmHg	52 ± 11	51 ± 13	60 ± 17	<0.01
Resistant high blood pressure, *n* (%)	6 (12.0)	12 (24.5)	19 (38.0)	<0.05
TBW_BIA_, L (% in BW)	30.1 ± 7.5	30.9 ± 7.2	32.7 ± 5.6	0.14
	(49.6 ± 5.6)	(55.3 ± 5.6)	(58.7 ± 5.1)	(<0.001)
ICW_BIA_, L (% in BW)	18.4 ± 4.7	18.8 ± 4.6	19.7 ± 3.4	0.30
	(30.2 ± 3.5)	(33.5 ± 3.8)	(35.2 ± 3.2)	(<0.001)
ECW_BIA_, L (% in BW)	11.7 ± 2.8	12.1 ± 2.6	13.1 ± 2.2	0.02
	(19.3 ± 2.1)	(21.7 ± 2.0)	(23.4 ± 2.1)	(<0.001)
Serum creatinine, mg/dL	1.67 ± 1.17	1.76 ± 1.27	2.55 ± 1.78	<0.01
eGFR_CKD-EPI_, ml/min per 1.73 m^2^	71.7 ± 48.8	71.0 ± 52.7	48.9 ± 46.2	<0.05
Serum albumin, g/dL	4.1 ± 0.4	4.0 ± 0.4	3.7 ± 0.5	<0.001
Total cholesterol, mg/dL	200 ± 34	193 ± 36	191 ± 55	0.53
Triglycerides, mg/dL	147 ± 71	139 ± 95	129 ± 88	0.55
Fasting blood glucose, mg/dL	103 ± 19	132 ± 45	134 ± 42	<0.05
UPCR, g/g · Cr	0.9 ± 1.5	0.8 ± 1.3	1.6 ± 1.9	<0.05
Uric acid, >7.0 mg/dL in men or >6.0 mg/dL in women, *n* (%)	23 (46.9)	27 (55.1)	32 (64.0)	0.23
24-hour urinary urea nitrogen excretion, g/day	2.1 ± 1.0	2.5 ± 1.9	2.2 ± 1.5	0.40
24-hour urinary creatinine excretion, g/day	1.5 ± 1.1	1.6 ± 1.5	1.5 ± 0.8	0.81
24-hour urinary sodium excretion, mmol/day	143 ± 58	128 ± 50	130 ± 54	0.37
Furosemide, *n* (%)	5 (10.0)	6 (12.2)	18 (36.0)	<0.01
Other diuretics, *n* (%)	7 (14.0)	7 (14.3)	6 (12.0)	0.94
ACE inhibitors, *n* (%)	14 (28.0)	17 (34.7)	16 (32.0)	0.77
AT1-R blockers, *n* (%)	30 (60.0)	28 (57.1)	25 (50.0)	0.58
Other antihypertensives	20 (40.0)	25 (51.0)	31 (62.0)	0.09

*Abbreviations*: *TBW*_*BIA*_ total body water as measured by bioimpedance analysis, *ICW*_*BIA*_ intracellular water as measured by bioimpedance analysis, *ECW*_*BIA*_ extracellular water as measured by bioimpedance analysis, *TBW*_*Watson*_ total body water calculated using the Watson formula, *eGFR*_*CKD-EPI*_ estimated glomerular filtration rate using the Chronic Kidney Disease Epidemiology Collaboration equation, *UPCR* urinary protein-to-creatinine ratio, *ACE inhibitors* angiotensin-converting enzyme inhibitors, *AT1-receptor blockers* angiotensin II type 1 receptor blockers.

### Independent factors associated with extracellular volume status

Age, male sex, diabetes mellitus, the eGFR, and the UPCR level were correlated with the %ECW_BIA_/TBW_Watson_ ratio in univariate analysis. In the multivariate analysis, male sex and UPCR remained independently associated with the %ECW_BIA_/TBW_Watson_ ratio (Table 2).

**Table 2:** **Independent factors associated with the percentage of ECW**_**BIA**_ **to TBW**_**Watson**_

Variables	Univariate analysis		Multivariate analysis*	
	ß (95% CI)	***P***-value	ß (95% CI)	***P***-value
Age, per 10 years of age	0.18 (0.04–0.72)	0.03	0.01 (-0.40–0.44)	0.94
Men	0.21 (0.17–1.27)	0.01	0.18 (0.04–1.16)	0.04
Diabetes mellitus	0.30 (0.67–2.13)	<0.001	0.11 (-0.31–1.31)	0.23
eGFR_CKD-EPI_, ml/min per 1.73 m^2^	-0.24 (-5.71 to -1.11)	0.01	-0.05 (-0.02–0.01)	0.63
UPCR, g/g · Cr	0.32 (0.33–0.96)	<0.001	0.17 (0.20–0.87)	0.01

*Note*: *Significant factors associated with the percentage of extracellular water volume as measured by bioimpedance analysis to total body water volume calculated by the Watson formula identified in the univariate analysis (*P* < 0.10) were subjected to multivariable analysis. When the eGFR calculated by the revised formula for Japanese patients based on the Modification of Diet in Renal Disease method was used as the dependent variable instead of eGFR_CKD-EPI_, the results remained similar (data not shown). Additionally, when the serum albumin level was used as the dependent variable instead of UPCR, the independent factors were sex, diabetes mellitus, and serum albumin (data not shown).

*Abbreviations*: *ECW*_*BIA*_ extracellular water as measured by bioimpedance analysis, *TBW*_*Watson*_ total body water calculated using the Watson formula, *eGFR*_*CKD-EPI*_ estimated glomerular filtration rate using the Chronic Kidney Disease Epidemiology Collaboration equation, *UPCR* urinary protein-to-creatinine ratio, *β* standardized regression coefficient, *CI* confidence interval.

### Correlation between serum albumin and extracellular volume status

As shown in Additional file 3: Figure S2 (online), the serum albumin level was weakly correlated with the %ECW_BIA_ in body weight (*r* = -0.32, *P* < 0.001) and ECW_BIA_/ECW_Peters_ ratio (*r* = -0.24, *P* = 0.004) and was moderately correlated with the %ECW_BIA_/TBW_Watson_ ratio (*r* = -0.44, *P* < 0.001).

### Correlations of %ECW_BIA_/TBW_Watson_ with renal outcome and all-cause mortality

**Figure 3:**
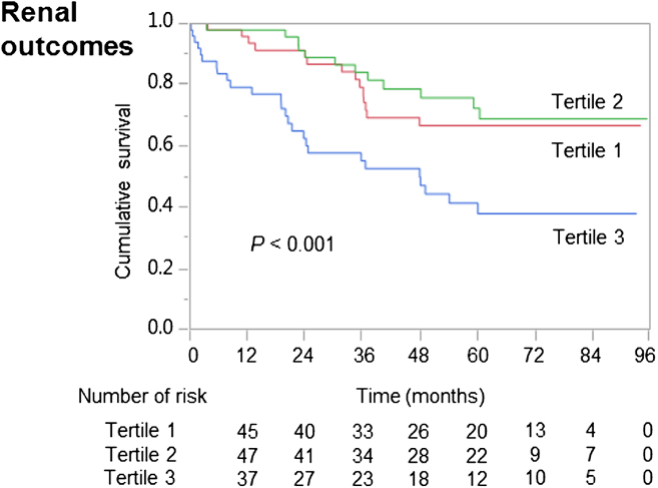
**Kaplan–Meier survival curves for adverse renal outcomes by tertiles of %ECW**_**BIA**_ **/TBW**_**Watson**_ **.**

During the follow-up period, 52 patients had adverse renal outcomes (8.5 per 100.0 patient years) and 25 had all-cause death (4.6 per 100.0 patient years). The Kaplan–Meier analysis curves revealed significant differences in renal outcomes among the different %ECW_BIA_/TBW_Watson_ tertiles (Figure 3). Patients in the highest tertile were at greater risk of disease progression (16.6 per 100.0 patient years) than were those in the lowest (8.1 per 100.0 patient years) and second tertiles (5.6 per 100.0 patient years) (*P* = 0.01). After adjustment for covariates including age, sex, diabetes mellitus, systolic blood pressure, UPCR level, and baseline eGFR, the %ECW_BIA_/TBW_Watson_ ratio was found to be significantly associated with adverse renal outcomes (HR, 1.21; 95% CI, 1.10–1.34; *P* < 0.001) (Table 3). As shown in Additional file 4: Figure S3 (online), we also depicted the Kaplan–Meier analysis curves for renal outcomes among the different tertiles of %ECW_BIA_ in body weight and ECW_BIA_/ECW_Peters_ ratio. Patients in the highest %ECW_BIA_/ECW_Peters_ tertile exhibited significantly lower renal survival than did the other two groups. In contrast, no significant differences were noted in renal survival according to the different tertiles of %ECW_BIA_ in body weight or tertiles 1 or 2 of %ECW_BIA_/ECW_Peters_ (*P* = 0.10).

**Table 3:** **Hazard ratios of different clinical variables for adverse renal outcomes**

Variables	Univariate analysis		Multivariate analysis*	
	HR (95% CI)	***P***-value	HR (95% CI)	***P***-value
Age, per 10 years of age	1.03 (1.01–1.05)	0.01	0.84 (0.69–1.04)	0.10
Men	1.82 (1.04–3.29)	0.04	1.13 (0.60–2.10)	0.70
Diabetes mellitus	3.60 (1.90–6.50)	<0.001	1.49 (0.72–2.98)	0.28
%ECW_BIA_/TBW_Watson_	1.24 (1.13–1.35)	<0.001	1.21 (1.10–1.34)	<0.001
Systolic blood pressure, mmHg	1.02 (1.01–1.04)	<0.001	1.00 (0.99–1.02)	0.63
Baseline eGFR_CKD-EPI_, ml/min/1.73 m^2^	0.92 (0.90–0.94)	<0.001	0.96 (0.95–0.98)	<0.001
UPCR, g/gCr	1.29 (1.15–1.42)	<0.001	1.20 (1.01–1.38)	0.04

*Note*: ^*^Adjusted for age, male sex, diabetes mellitus, systolic blood pressure, ECW_BIA_/TBW_Watson_ percentage, baseline estimated glomerular filtration rate, and urinary protein–creatinine ratio (variables associated with adverse renal outcomes identified in the univariate analysis [*P* < 0.10] were entered into the multivariable model). When the ECW_BIA_/ECW_Peters_ ratio was used as the dependent variable instead of the %ECW_BIA_/TBW_Watson_ ratio, the results remained similar (data not shown).

*Abbreviations*: *HR* hazard ratio, *CI* confidence interval, *%ECW*_*BIA*_ */TBW*_*Watson*_ percentage of extracellular water as measured by bioimpedance analysis to total body water calculated using the Watson formula, *eGFR*_*CKD-EPI*_ estimated glomerular filtration rate using the Chronic Kidney Disease Epidemiology Collaboration equation, *UPCR* urinary protein-to-creatinine ratio.

### Prognostic performance of %ECW_BIA_/TBW_Watson_ ratio in predicting adverse renal outcomes

**Figure 4:**
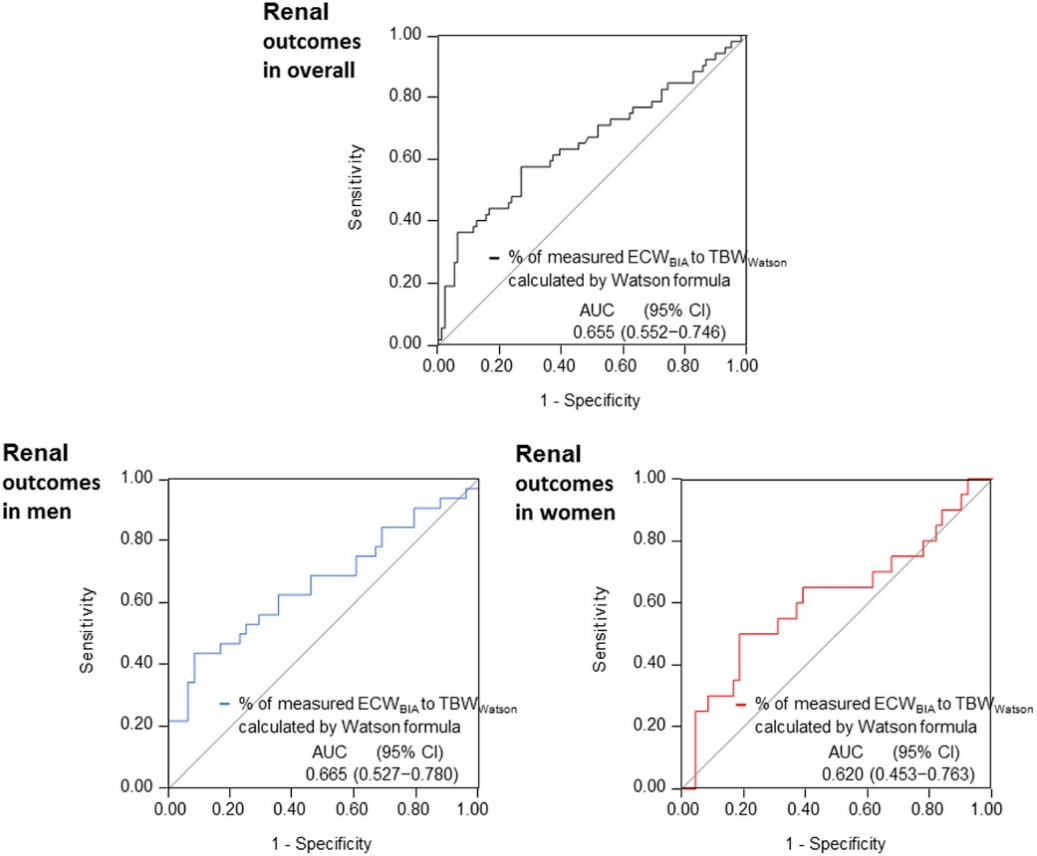
**Receiver operating characteristic curves in the assessment of %ECW**_**BIA**_ **/TBW**_**Watson**_ **as a prognostic factor of adverse renal outcomes in all patients, male patients, and female patients.** Abbreviations: TBW_BIA_, total body water as measured by bioimpedance analysis; TBW_Watson_, total body water calculated using the Watson formula; AUC, area under the curve; CI, confidence interval.

We constructed receiver operating characteristic curves to determine the cut-off of the %ECW_BIA_/TBW_Watson_ ratio that best predicts adverse renal outcomes. The optimal cut-off values for all patients, male patients, and female patients were 39.9%, 42.2%, and 40.0%, respectively. Using these cut-off values, the respective areas under the curve were 0.655 (95% CI, 0.552–0.746), 0.665 (95% CI, 0.527–0.780), and 0.620 (95% CI, 0.453–0.763) (Figure 4).

## Discussion

This study revealed that the ECW_BIA_/TBW_Watson_ ratio reflects the extracellular volume status and is associated with adverse renal outcomes in patients with CKD. Additionally, our findings showed that the %ECW_BIA_/TBW_Watson_ ratio is influenced by sex and proteinuria. Finally, age affected the balance between the ICW and ECW and increased the percentage of ECW in the body fluid composition.

BIA methods are used to noninvasively measure the ECW, ICW, and TBW and calculate the free fat mass and fat based on empirical equations [19]. However, the measure of free fat mass is influenced by the TBW content because the fat free mass is obtained by dividing the TBW by 0.733. The ECW_BIA_/TBW_BIA_ ratio increases along with ECW excess and decreased ICW. Therefore, the ECW_BIA_/TBW_BIA_ ratio appears to be an inadequate marker of the volume status [7]. In fact, the present findings show that the percentage of ECW_BIA_ in TBW_BIA_ was positively correlated with age. Two recent studies used a new BIA device to assess the volume status [8, 20]. The device quantified fluid overload using reference values derived from the pooled data of healthy subjects [21]. We preliminarily used the expected ECW as calculated by the Peters formula and the expected TBW as calculated by the Watson formula as reference values of body fluid composition. These two parameters were highly correlated with each other in our study population (*r* = 0.97 for men and *r* = 0.98 for women) (data not shown). Peters et al. reported that the ECW was retained in healthy potential donors of older ages [10]. On the other hand, the Watson formula includes age in men as a coefficient. The measured ECW_BIA_ gradually decreased with age in our study. As a result, elderly patients tended to exhibit a lower ECW_BIA_/ECW_Peters_ ratio. The ECW_BIA_/ECW_Peters_ ratio may be not a useful maker if the extracellular volume changes with age only in patients with CKD and not in healthy subjects. The present findings show that the ratio of the measured extracellular volume to the estimated body fluid volume can serve as a useful marker of the volume status in patients with CKD. This result may be one reason that the %ECW_BIA_/TBW_Watson_ ratio exhibited a relative increase in the extracellular volume with respect to the expected fluid status, resulting in a good balance between the ICW and ECW for age. Notably, a higher extracellular volume was associated with adverse renal outcomes during a relatively long follow-up period (median duration of 4.9 years) for both of the ECW_BIA_/ECW_Peters_ ratio and the %ECW_BIA_/TBW_Watson_ ratio. Additionally, the ECW_BIA_/TBW_Watson_ ratio was associated with traditional risk factors for kidney disease progression, including age, male sex, diabetes mellitus, higher pulse pressure, resistant hypertension, lower eGFR, lower serum albumin level, and higher proteinuria level. These findings may suggest that a higher extracellular volume causes hemodynamic instability. In contrast, sodium excretion was not associated with fluid excess. Whether sodium intake and sodium retention have a one-to-one relationship and whether sodium intake is linearly associated with the development of end-stage renal disease remain unclear [22–24]. Younger subjects and those without uremia consume a higher variety of foods with various levels of sodium, protein, and total calories than do elderly people and patients with uremia. These biases may have hampered studies in this area.

Proteinuria was a strong factor in the development of a higher extracellular volume. Hypoalbuminemia, mostly in association with massive proteinuria, produces an increased interstitial fluid volume and a contracted intravascular volume contraction by a diminished oncotic pressure gradient, thus inducing renal sodium retention by activation of the renin-angiotensin-aldosterone system [25]. In addition, differences in all parameters of the extracellular volume status were found between men and women; specifically, it the extracellular volume was lower in women. The ECW and ICW contents are generally lower in women than in men because women have a relatively higher proportion of fat. Otherwise, differences between these two parameters may be caused by inaccuracies in the determination of the measured fluid content and estimated fluid content. Our findings revealed a difference between the measured TBW_BIA_ and estimated TBW_Watson_ in both men and women (Additional file 2: Figure S1). In both sexes, these differences increased with increases in the measured TBW_BIA_ (Additional file 2: Figure S1B and D). Thus, differences between the actual values and reference values may contribute to the fluid volume status. The Watson formula was designed to estimate the TBW of healthy Caucasian subjects; however, it must be interpreted with caution because it may overestimate the TBW_Watson_ in Japanese patients, especially women. A previous study reported differences in the TBW among individuals of different races [26].

Aging cells are known to shrink and undergo apoptosis [27–29]. Fluid imbalance in patients with CKD is characterized by excess ECW associated with sodium retention [30, 31] and decreased ICW associated with malnutrition [32]. In fact, patients with CKD with a leaner body mass have a higher prevalence of hypertension, poorer control of hypertension, and greater incidence of left ventricular hypertrophy. This phenomenon is known as the "obesity paradox" [33, 34]. Our findings highlight the notion that elderly patients with CKD may be more susceptible to volume overload because the reduced intracellular volume caused by aging and malnutrition lessens the capacity of cells to retain fluid. This strongly supports the hypothesis that a lower serum albumin level is significantly correlated with the fluid volume.

The best %ECW_BIA_/TBW_Watson_ ratio cut-offs for adverse renal outcomes among male, female, and all patients were 42.2%, 40.0%, and 39.9%, respectively. These values were close to the ECW/ICW ratio of 2:3. The abovementioned threshold values may be useful for determining extracellular volume excess. However, whether removal of excess fluid improves renal outcomes remains unclear.

This study has several limitations. First, it was a retrospective cohort study conducted at a single center. However, it provided detailed information on patients’ body fluid composition and had a relatively long follow-up period. Second, the ECW_BIA_/TBW_Watson_ ratio may not be a precise indicator of volume status; the estimated TBW_Watson_ and actual TBW_BIA_ differ in Japanese patients. We recognize that this parameter was not compared with an indicator for congestive heart failure, such as the N-terminal of the prohormone brain natriuretic peptide. Regardless, the ECW_BIA_/TBW_Watson_ ratio is associated with many factors associated with fluid volume overload; BIA can be used to easily and noninvasively assess the body fluid composition, and the results correlate with those of isotopic dilution and dual-energy X-ray absorptiometry [35]. The estimated TBW_Watson_ is widely used in this area. We believe that the ECW_BIA_/TBW_Watson_ ratio is a practical parameter of the extracellular volume status and that the reported data will support future studies in this area.

## Conclusions

In the present study, the ECW_BIA_/TBW_Watson_ ratio was associated with adverse renal outcomes during a relatively long follow-up period. This finding suggests that a higher extracellular volume has an adverse effect on kidney disease. Proteinuria is independently associated with the extracellular volume status. Aging leads to change in the balance between the ICW content and ECW content and increases the percentage of the ECW content in the body fluid composition. Consequently, elderly patients with CKD may be susceptible to volume overload.

## Electronic supplementary material

Additional file 1: Table S1: Correlations between demographic characteristics and parameters of extracellular volume status. (DOC 38 KB)

Additional file 2: Figure S1: Agreement between total body water as measured by bioimpedance analysis and total body water calculated using the Watson formula. Abbreviations: TBW_BIA_, total body water as measured by bioimpedance analysis; TBW_Watson_, total body water calculated using the Watson formula; CI, confidence interval. (TIFF 1 MB)

Additional file 3: Figure S2: Correlations between serum albumin level and %ECW_BIA_ in body weight and %ECW_BIA_/TBW_Watson_. Abbreviations: ECW_BIA_, extracellular water; TBW_Watson_, total body water calculated using the Watson formula. (TIFF 1 MB)

Additional file 4: Figure S3: Kaplan–Meier survival curves for adverse renal outcomes by (A) tertiles of %ECW_BIA_ in body weight and (B) ratio of ECW_BIA_ to ECW_Peters_. Abbreviations: ECW_BIA_, extracellular water; BW, body weight; TBW_BIA_, total body water as measured by bioimpedance analysis; TBW_Watson_, total body water calculated using the Watson formula. (TIFF 985 KB)

## Abbreviations

CKD: Chronic kidney disease
ECW: Extracellular water
TBW: Total body water
ICW: Intracellular water
BIA: Bioimpedance analysis
BMI: Body mass index
Cr: Serum creatinine
eGFR: Estimated glomerular filtration rate
UPCR: Urinary protein-to-creatinine ratio
HR: Hazard ratio
CI: Confidence interval.
