# Supplementary material for: Association between ratio of measured extracellular volume to expected body fluid volume and renal outcomes in patients with chronic kidney disease: a retrospective single-center cohort study
Source: BMC Nephrol. 2014 Dec 1;15:189. doi: 10.1186/1471-2369-15-189 (PMC4268815; doi:10.1186/1471-2369-15-189)
Supplement: Supplementary file 1 — Additional file 1: Table S1: Correlations between demographic characteristics and parameters of extracellular volume status. (DOC 38 KB) [file 12882_2014_880_MOESM1_ESM.doc]

**Additional file 1: Table S1:** Correlations between demographic characteristics and parameters of extracellular volume status

| Demographic characteristics | Tertiles of %ECW_BIA_ in body weight | | Tertiles of ECW_BIA_ to ECW_Peters_ | | Tertiles of %ECW_BIA_ to TBW_Watson_ | |
| --- | --- | --- | --- | --- | --- | --- |
|  | ß | *P* | ß | *P* | ß | *P* |
| Age | −0.066 | 0.42 | −0.133 | 0.11 | 0.130 | 0.11 |
| Height | 0.093 | 0.261 | 0.248 | <0.01 | 0.097 | 0.24 |
| Weight | −0.447 | <0.001 | −0.094 | 0.25 | −0.158 | 0.05 |
| Body mass index | −0.645 | <0.001 | −0.296 | <0.001 | −0.272 | <0.001 |
| Diabetes mellitus | 0.026 | 0.75 | 0.116 | 0.16 | 0.201 | <0.05 |
| Resistant high blood pressure | 0.043 | 0.60 | 0.195 | <0.05 | 0.247 | <0.01 |
| eGFR_CKD-EPI_ | −0.050 | 0.37 | −0.016 | 0.846 | −0.187 | <0.05 |
| Serum albumin | −0.253 | <0.01 | −0.201 | <0.05 | −0.319 | <0.001 |
| UPCR | 0.130 | 0.12 | 0.195 | <0.05 | 0.169 | <0.05 |
| Furosemide | 0.046 | 0.58 | 0.170 | <0.05 | 0.269 | <0.001 |
| Other antihypertensives | −0.041 | 0.62 | 0.041 | 0.62 | 0.180 | <0.05 |

Abbreviations: TBW_BIA_, total body water as measured by bioimpedance analysis; ECW_BIA_, extracellular water as measured by bioimpedance analysis; ECW_Peters_, extracellular water calculated using the Peters formula; TBW_Watson_, total body water calculated using the Watson formula; eGFR_CKD-EPI_, estimated glomerular filtration rate using the Chronic Kidney Disease Epidemiology Collaboration equation; UPCR, urinary protein to creatinine ratio
