# Supplementary figures and images for: Association between ratio of measured extracellular volume to expected body fluid volume and renal outcomes in patients with chronic kidney disease: a retrospective single-center cohort study
Source: BMC Nephrol. 2014 Dec 1;15:189. doi: 10.1186/1471-2369-15-189 (PMC4268815; doi:10.1186/1471-2369-15-189)

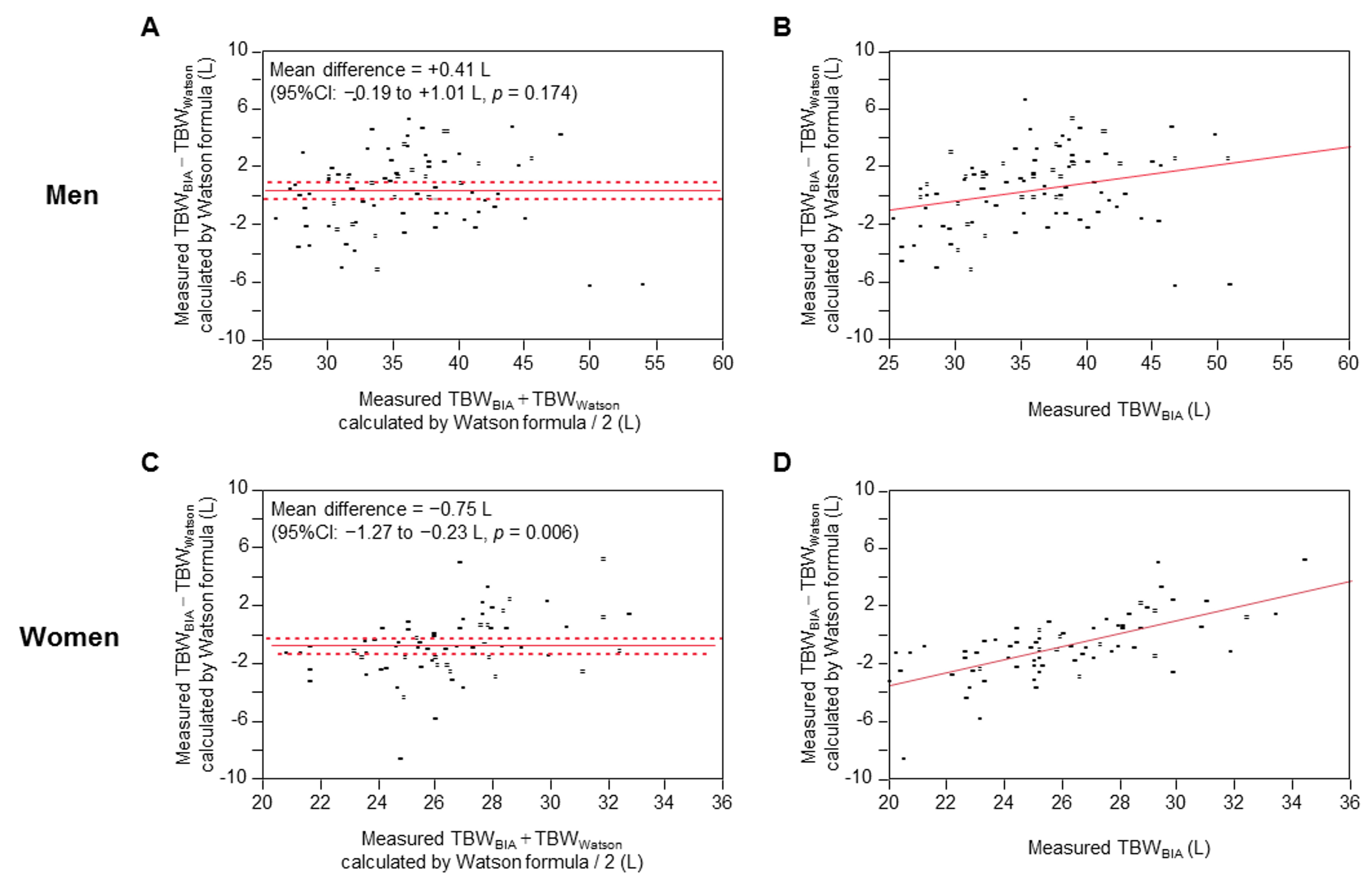

Supplement: Supplementary file 2 — Additional file 2: Figure S1: Agreement between total body water as measured by bioimpedance analysis and total body water calculated using the Watson formula. Abbreviations: TBWBIA, total body water as measured by bioimpedance analysis; TBWWatson, total body water calculated using the Watson formula; CI, confidence interval. (TIFF 1 MB) [file 12882_2014_880_MOESM2_ESM.tiff]

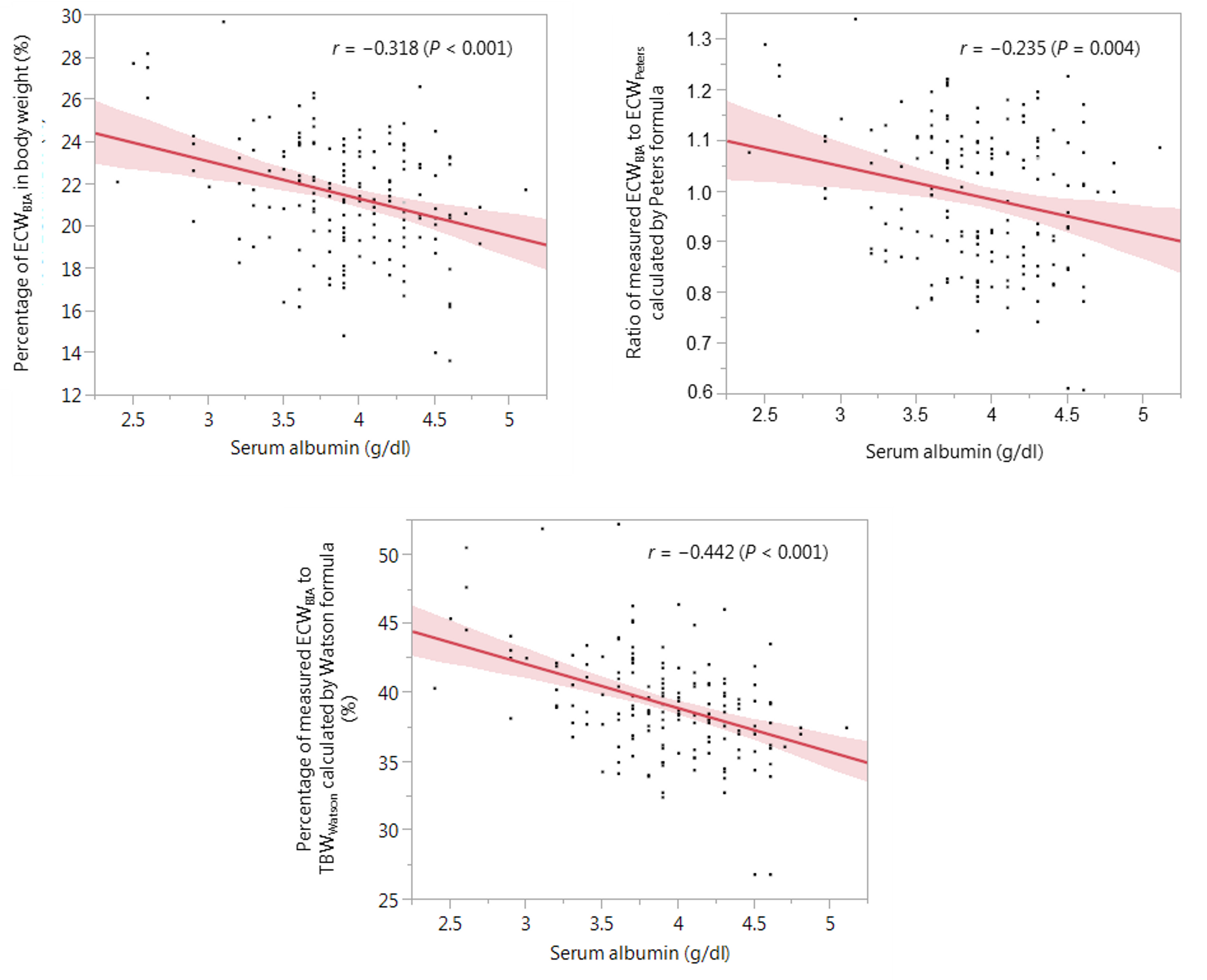

Supplement: Supplementary file 3 — Additional file 3: Figure S2: Correlations between serum albumin level and %ECWBIA in body weight and %ECWBIA/TBWWatson. Abbreviations: ECWBIA, extracellular water; TBWWatson, total body water calculated using the Watson formula. (TIFF 1 MB) [file 12882_2014_880_MOESM3_ESM.tiff]

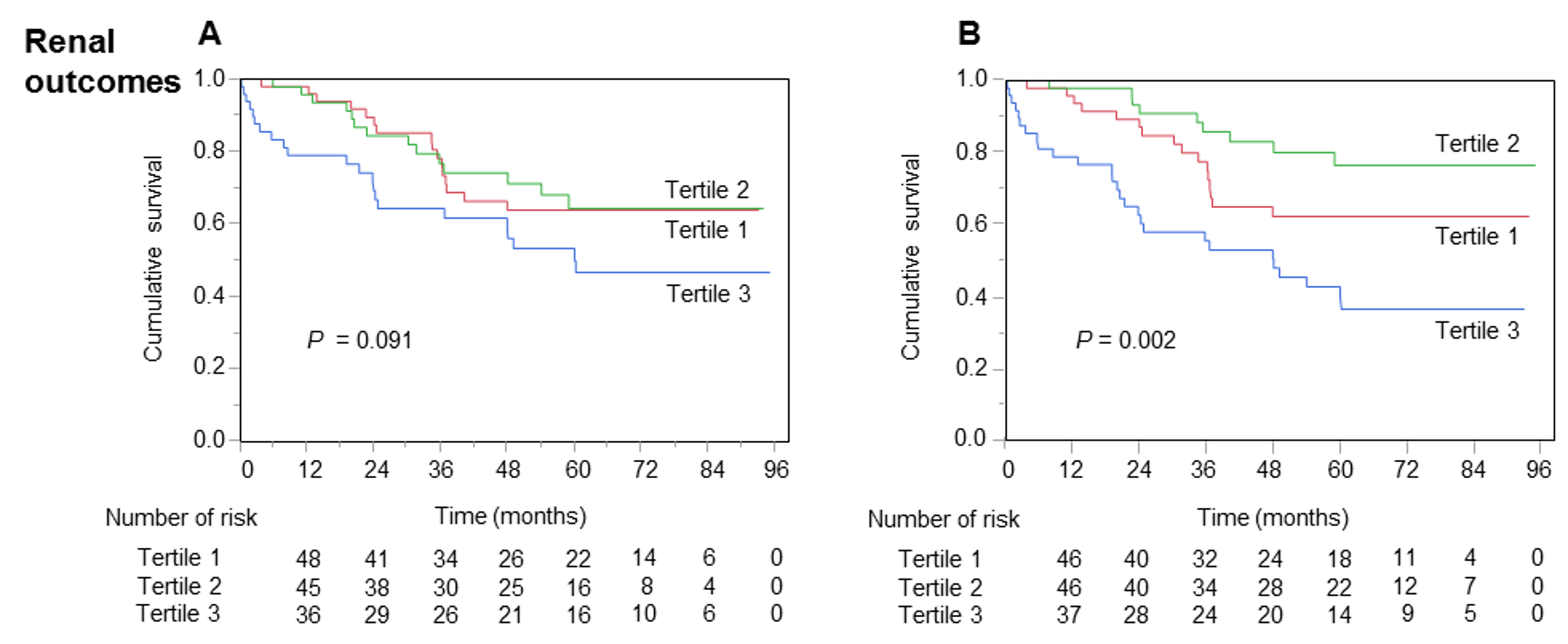

Supplement: Supplementary file 4 — Additional file 4: Figure S3: Kaplan–Meier survival curves for adverse renal outcomes by (A) tertiles of %ECWBIA in body weight and (B) ratio of ECWBIA to ECWPeters. Abbreviations: ECWBIA, extracellular water; BW, body weight; TBWBIA, total body water as measured by bioimpedance analysis; TBWWatson, total body water calculated using the Watson formula. (TIFF 985 KB) [file 12882_2014_880_MOESM4_ESM.tiff]
